# Mechanically Flexible, Large-Area Fabrication of Three-Dimensional Dendritic Au Films for Reproducible Surface-Enhanced Raman Scattering Detection of Nanoplastics

**DOI:** 10.1021/acssensors.4c02081

**Published:** 2024-10-31

**Authors:** Rafael Villamil Carreón, Ana G. Rodríguez-Hernández, Laura Elvira Serrano de la Rosa, José Juan Gervacio-Arciniega, Siva Kumar Krishnan

**Affiliations:** † Facultad de Ciencias Físico Matemáticas, Benemérita Universidad Autónoma de Puebla, Av. San Claudio y Av. 18 sur., Puebla, Pue., C.P. 72570, México; ∥ CONAHCyT-Centro de Nanociencias and Nanotecnología, Universidad Nacional Autónoma de México, Km 107 Carretera Tijuana-Ensenada, Ensenada, Baja California C.P. 22800, México; $ Instituto de Física, Benemérita Universidad Autónoma de Puebla, Apdo. Postal J-48, Puebla, Pue. 72570, México; § CONAHCyT- Facultad de Ciencias Físico Matemáticas, Benemérita Universidad Autónoma de Puebla, Apdo. Postal J-48, Puebla 72570, México; # CONAHCyT-Instituto de Física, Benemérita Universidad Autónoma de Puebla, Apdo. Postal J-48, Puebla, Pue. 72570, México

**Keywords:** thermal evaporation, deep eutectic solvent, dendritic Au film, 3D SERS substrate, nanoplastics detection

## Abstract

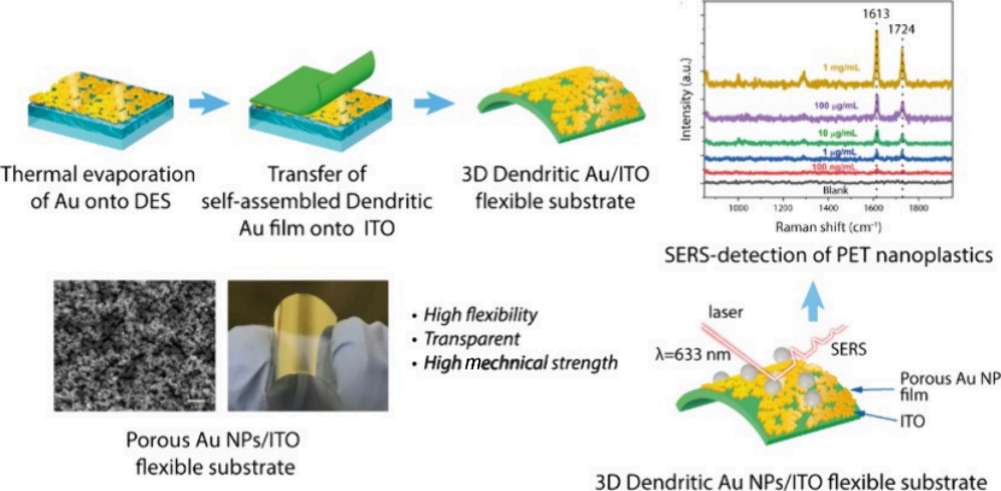

The escalating crisis of nanoplastic pollution in water and food products demands the development of novel methodologies for detection and recycling. Despite various techniques available, surface-enhanced Raman scattering (SERS) is emerging as a highly efficient technique for the trace detection of micro/nanoplastics. However, the development of highly reproducible and stable, flexible SERS substrates that can be used for sensitive detection in environmental medium remains a challenge. Here, we report a fabrication of large-area, three-dimensional (3D), and highly flexible SERS substrate based on porous dendritic Au films onto a flexible indium tin oxide (ITO) substrate via facile, thermal evaporation of Au over the vacuum-compatible deep eutectic solvent (DES)-coated glass substrate and subsequent direct transfer process. The as-fabricated 3D dendritic Au/ITO flexible substrates can be used for ultrasensitive SERS detection of crystal violet (CV) as probe analyte molecules with the limit of detection (LOD) as low as 6.4 × 10^–15^ M, with good signal reproducibility (RSD of 11.3%). In addition, the substrate showed excellent sensitivity in detecting nanoplastics such as poly(ethylene terephthalate) (200 nm) and polystyrene (100 nm) with LODs reaching up to 0.051 and 8.2 μg/mL, respectively. This work provides a facile approach for the preparation of highly flexible plasmonic substrates, showing great potential for the SERS detection of a variety of environmental pollutants.

Excessive use of plastics in everyday life has led to serious environmental pollution generated by the degradation of plastic into microplastics (1 μm to 5 mm), which has become a critical concern, mainly because of their risk to diverse ecosystems and human health.^1,2^ The nanoplastics are distinguished from microplastic particles concerning their smaller size (<1 μm), high surface reactivity, stability, mobility, and toxicity upon environmental exposure, causing severe environmental pollution.^3^ This nanoplastic pollution is primarily generated by the ubiquitous occurrence of microplastic fragmentation into smaller particles with relatively smaller sizes, and its effects on global pollution is poorly understood.^4,5^ Given their substantial low concentration, varied particle size and shapes, and slow degradation rate, nanoplastics consequently present a challenge for standardized detection and monitoring to prevent the threat to the environment.^6,7^ Additionally, the abundance of nanoplastics is much higher than that of the microplastics in the environment, which raises toxicological concerns in human health through inhalation,^8^ bioaccumulation in food packages,^9^ and consumption of contaminated drinking water^10,11^ and milk products.^12^ Therefore, detecting these nanoplastic particles holds paramount research importance in mitigating environmental pollutants caused by them.

Numerous methods have been developed such as Fourier transform infrared spectroscopy,^13^ Raman spectroscopy,^14^ dark-field hyperspectral microscopy,^15^ impedance spectroscopy,^16^ and microfluidic techniques^17^ for detection of micro/nanoplastics. Despite several methods developed for detecting microplastic particles, their sensitivity is considerably limited due to their small particle size and low concentration in the environment and food products. Recently, surface-enhanced Raman spectroscopy (SERS)^18^ has emerged as a nondestructive, cost-effective, and highly sensitive detection technique for micro/nanoplastics in complex environments.^19,20^ The SERS substrates, particularly those made of plasmonic nanoparticles such as Ag, Au, and Cu nanoparticles (NPs), are recognized for their high sensitivity owing to localized surface plasmon resonance (LSPR) and strong electromagnetic (EM) field distribution in the gaps (less than 10 nm) between adjacent NPs.^21,22^ The EM field confinement at the nanometric gaps is several orders higher, so-called “hotspots”, which allows amplifying the SERS intensity of analyte molecules with an enhancement factor above 10^8^.^6,23^ However, it is challenging to functionalize larger analyte molecules such as micrometer-sized nano/microplastics over the conventional metal NP surfaces due to the fact that only a small cross section of the particle is excited by the local electric field.^14^ Moreover, these substrates exhibit minimum capillary forces and less sensitivity, which limit their capacity to identify micro/nanoparticles in environmental samples.^24^ Alternatively, creating a three-dimensional (3D) nanostructure is considered to be one of the promising approaches for increasing “hotspots” and trapping micro/nanoplastics within the pore sites for sensitive SERS detection.^25−27^ Numerous synthetic techniques such as electron-beam lithography or photolithography,^28^ nanoimprint lithography,^29^ 3D printing,^27^ or conventional colloidal self-assembly process^30,31^ have been explored to create a high density of “hotspots”. Nevertheless, these techniques are time-consuming and costly.

Recently, the development of a highly flexible SERS substrate by depositing plasmonic nanoparticles over highly flexible and transparent substrates has been emerging for sensitive detection of a variety of organic molecules, contaminations in a complex environment, and point-of-care diagnostics.^32^ Compared with conventional rigid SERS substrates, highly flexible materials such as polymer films,^33^ hydrogels,^34^ graphene/MXene fibers,^35^ cellulose paper,^36^ adhesive tape,^37^ nanomica,^38^ commercial digital versatile discs (DVDs),^39^ etc., have been demonstrated to exhibit intimate contact with the different surfaces, providing opportunities in rapid analysis with high sensitivity. The majority of the earlier works demonstrated the assembly of plasmonic NPs over highly transparent and flexible substrates including poly(dimethylsiloxane) (PDMS),^40^ polyimide (PI) films,^41^ polycarbonates (PC),^42^ and silicon rubber,^43^ which showed impressive performance in direct analysis of pesticide residues from fruit and vegetable peels since they have a low scattering cross section and do not affect the Raman signal of the sample. For instance, previous research has demonstrated that the formation of 3D Ag nanofoam nanostructures can generate more volumetric-like plasmon modes and can efficiently trap microplastic particles, resulting in enhanced SERS sensitivity for microplastic detection.^26^ Zhu et al.^44^ recently proposed a photoinduced enhanced Raman scattering technique using Ag/ZnO@PDMS nanorod arrays for the detection of microplastics of larger sizes (800 nm) in environmental samples. Moreover, few previous studies have reported the formation of cavity or porous metallic nanostructures to trap the micro- or nanoplastics to enhance the SERS signals.^45,46^ Despite these efforts, the formation of uniform 3D porous structures with high density and accessible “hotspots” for the detection of smaller nanoplastics in complex environmental samples remains a great challenge.

In this work, we demonstrate a facile, versatile, and low-cost synthetic approach for fabricating a highly flexible SERS substrate based on self-assembled dendritic Au films onto a flexible ITO substrate, which are deposited through vacuum thermal evaporation onto DES-coated substrates and subsequent transfer process. Deposition of Au wire under lower pressure (2 × 10^–4^ mbar) onto the DES substrate enables the formation of 3D dendritic Au films, in which the use of DES on the growth substrate plays a key role in the self-assembly of deposited Au NPs and generates porous dendritic-like Au films. The as-fabricated flexible SERS substrate exhibited high SERS sensitivity down to 6.4 × 10^–15^ M for detecting probe molecules (crystal violet (CV)), along with excellent signal reproducibility. Moreover, the flexible SERS substrate demonstrated exceptional sensitivity for the trace detection of poly(ethylene terephthalate) (PET) and polystyrene (PS) nanoplastics, even in real environmental and food samples such as tap water, lake water, and diluted milk and wine samples.

## Experimental Section

### Materials

Choline chloride (HOC_2_H_4_N[CH_3_]_3_Cl, ≥98%), urea (NH_2_CONH_2_, 99.0%), PS particles of 100 nm in size, and indium tin oxide (ITO)-coated PET flexible substrate were procured from Merck Mexico. Gold wire (100%, 0.2 mm) was sourced from SPI Supplies. Acetone, trichloroethylene, and ethyl alcohol were obtained from J. T. Baker. All chemicals, certified for analytical purity, were utilized without further preparation.

### Preparation of DES

The ChCl/urea-based DES was prepared using a previously reported procedure.^47^ Initially, choline chloride (ChCl) was dried completely at 90 °C to ensure optimal conditions for DES preparation. Subsequently, ChCl was combined at a 1:2 molar ratio with urea (U). The resulting mixture was heated in an oven at 90 °C until a clear, homogeneous liquid was attained. After being cooled to ambient temperature, the DES was stored for further thermal deposition processes.

### Preparation of Flexible 3D Dendritic Au/ITO Films

**Scheme 1:**
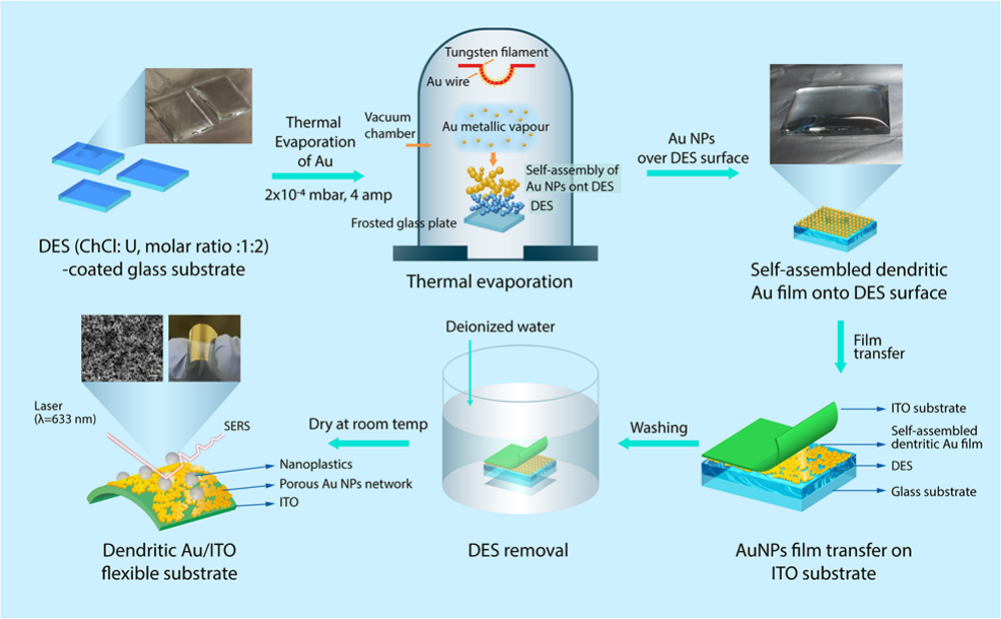
Schematic Diagram of the Fabrication Procedure of the Self-Assembled Porous Au NPs/ITO Flexible Substrate for SERS Detection of Nanoplastics

A cleaned frosted glass slide employed in this study underwent thorough cleaning in acetone, trichloroethylene, and ethyl alcohol in an ultrasonic bath, each for 10 min. Subsequently, a DES was coated onto the frosted glass surface with a thickness of ∼1 mm, covered an area of 2.5 × 2.5 cm^2^, and were placed inside the vacuum evaporation chamber. After that, Au wires were thermally evaporated onto the DES-coated surface at a pressure of 2 × 10^–4^ mbar. All samples were subjected to a 4 A current supplied to the tungsten filament during deposition. After the deposition, the samples were held in a vacuum for 5 min and then removed from the evaporation chamber. Subsequently, the Au film then adhered gently to the clean flexible ITO substrate to form a sandwich structure (Scheme 1). Finally, the self-assembled dendritic Au NP film-coated ITO substrates were carefully washed by spraying with DI water to eliminate DES residues, and the sample was dried at room temperature.

### FDTD Simulation of the 3D Au Film

To gain deeper insights into the electric field distribution in dendritic Au films, three-dimensional finite-difference time-domain (3D-FDTD)^48,49^ simulations were conducted. The FDTD solutions software (Lumerical Inc., Canada version) facilitated these optical simulations. The simulations employed a total-field/scattered-field (TFSF) source with perfectly matched layer (PML) boundary conditions. The structural parameters for the FDTD model were derived from atomic force microscopy (AFM) and scanning electron microscopy (SEM) images. The simulation region was established at about 1 × 1 μm^2^, and the refractive index of the surrounding medium was set to 1.0 to replicate air conditions.

### Material Characterization

SEM images were acquired using a JEOL-JSM7401F field emission scanning electron microscope operating at 15 kV. Topography images were obtained using the XE-7 Park Systems Atomic Force Microscopy instrument (Park Systems Corp., Suwon, Korea). Room-temperature SERS spectra of the porous Au/ITO films were recorded using a Bruker (SENTERRA) Raman spectrometer, with a 633 nm He–Ne laser as the excitation source and a laser power of 1.6 mW. The diameter of the exciting laser spot was consistently set at 4.7 μm under a 50× lens, and the signal acquisition time was fixed at 2 s for 3 cycles. The SERS mapping measurement was performed using the same equipment with a 633 nm laser. The area selected was 2 × 2 μm^2^ with a time interval of 0.1 s acquisition time, and the step size was set to 0.1 μm.

### SERS Measurements

For SERS detection of analyte molecules (CV), the porous Au/ITO substrates were cut into small pieces, and a 25 μL aqueous solution of CV molecules with different concentrations was dropped onto the substrates, followed by allowing to naturally dry at room temperature before acquiring the SERS spectra. For SERS detection of nanoplastics, the PET nanoplastics, with an average size of 200 nm, were synthesized following our previously reported protocol.^50^ The commercial monodisperse polystyrene (PS) nanospheres with a standard particle size of ∼100 nm were purchased from Merck, Mexico. The nanoplastic particles were dispersed in DI water at various concentrations under sonication for 1 h. After that, 25 μL of dispersed PET and PS nanoplastic particles was dropped onto the flexible porous Au/ITO substrates, and the substrates were left to dry at room temperature for 3 h. The SERS spectra were recorded by localizing the nanoplastic particles inside the porous dendritic Au/ITO film substrate.

### SERS Detection of PET NPs in Real Samples

In the pursuit of detecting nanoplastics in real samples, we employed four readily accessible environmental and natural beverages samples, such as tap water, pond water, and diluted milk and wine. For each sample, a 10 mL dilution was made by adding 1 mL of the PET and PS nanoplastic solution with varying concentrations to 9 mL of DI water. Subsequently, 25 μL of each dilution prepared with PET and PS nanospheres was then deposited onto the porous Au/ITO substrates, and the samples were permitted to air-dry at room temperature prior to conducting SERS measurements.

## Results and Discussion

### Characterization of the Porous Au NPs/ITO Flexible Substrate

The fabrication procedure of a flexible Au NPs/ITO substrate is briefly depicted in Scheme 1. First, the Au NP film is deposited onto a DES (ChCl:urea, molar ratio of 1:2)-coated glass substrate using thermal evaporation of Au wire with a pressure of 2 × 10^–4^ mbar and by applying a current of 4 A (Figure S1). Then, the Au film surface is gently brought into contact with a flexible indium tin oxide (ITO, area of 2 × 2 cm^2^) to form a sandwich-like structure. Subsequently, the DES is slowly removed by gentle washing cycles with deionized (DI) water, the deposited Au NP film on the ITO substrate is peeled off, and it is left to dry under ambient conditions to achieve a flexible Au/ITO substrate. By using the same procedure, the porous Au films can be transferred onto a flexible PEN substrate (Au/PEN). For comparison, the Au NPs were deposited over a glass substrate without coating DES on it. The results show that a uniform Au NP film with relatively smooth surfaces was observed (Figure S2), highlighting the crucial role of DES in the self-assembly and formation of the dendritic Au film.

**Figure 1:**
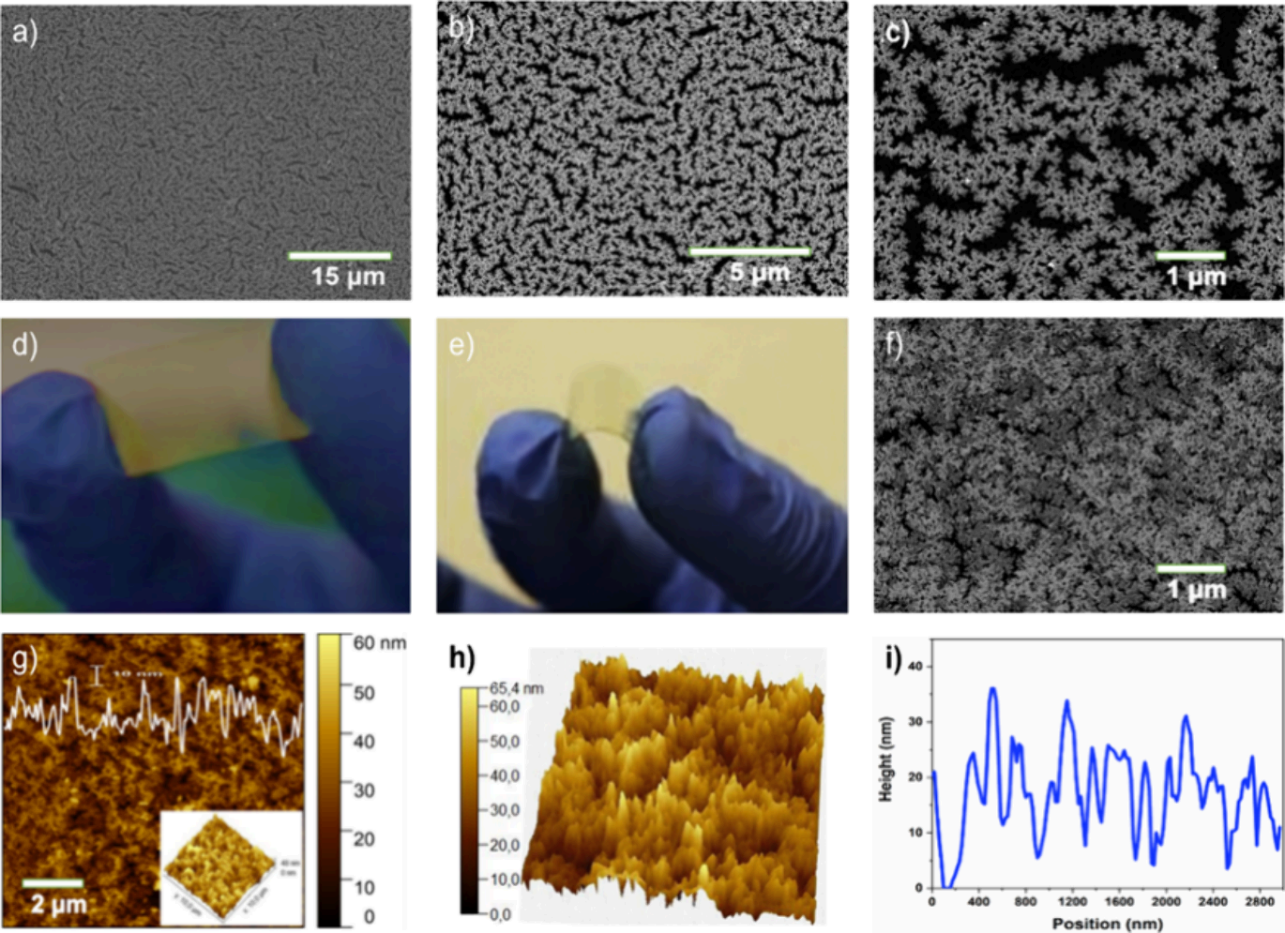
Morphological characterization of a dendritic Au film and Au/ITO substrate. (a–c) Typical SEM images of self-assembled dendritic Au films obtained at a pressure of 2 × 10^–4^ mbar. (d, e) Photographic images, (f) SEM image, (g, h) AFM topographical image, and (i) corresponding line profile plot of the 3D dendritic Au/ITO flexible substrate.

Figure 1a–c shows the morphological characterization of a 3D dendritic Au NP film deposited over a glass substrate. As shown in the SEM image (Figure 1a), the Au NP films consisting of dendritic-like Au nanostructures that are self-assembled into a 3D interconnected network structure with nanometric gaps were formed. The close-up SEM image (Figure 1b,c) clearly reveals a distinct branched dendritic 3D morphology with an average particle size of 12.16 ± 2.66 nm and an average pore size of 303.75 ± 45.3 nm (Figure S3). It is important to mention that deposition at higher pressure (1 × 10^–2^ mbar) leads to the formation of self-assembled Au NPs that evolved a 1D self-assembled chain-like structure (Figure S4), which indicates that lowering deposition pressure is a requisite for achieving a dendritic Au film with uniform and highly accessible “hotspots”, which is crucial for SERS signal enhancement.^51^Figure 1d,e shows the photographic images of the porous Au NP-supported flexible ITO substrate (Au/ITO), which reveals the stable and uniform deposition onto ITO flexible substrates. As shown in the SEM and AFM images (Figure 1f,g), the porous dendritic 3D structure is preserved over the ITO substrate. In addition, the AFM topographical image and corresponding height profile (Figure 1h,i) further evidence a porous Au film with uniform nanometric pores with heights ranging between 20 and 35 nm. These results indicate that the 3D self-assembled dendritic Au films were successfully transferred to the surface of the ITO substrate.

### SERS Performance of the 3D Dendritic Au/ITO Flexible Substrate

**Figure 2:**
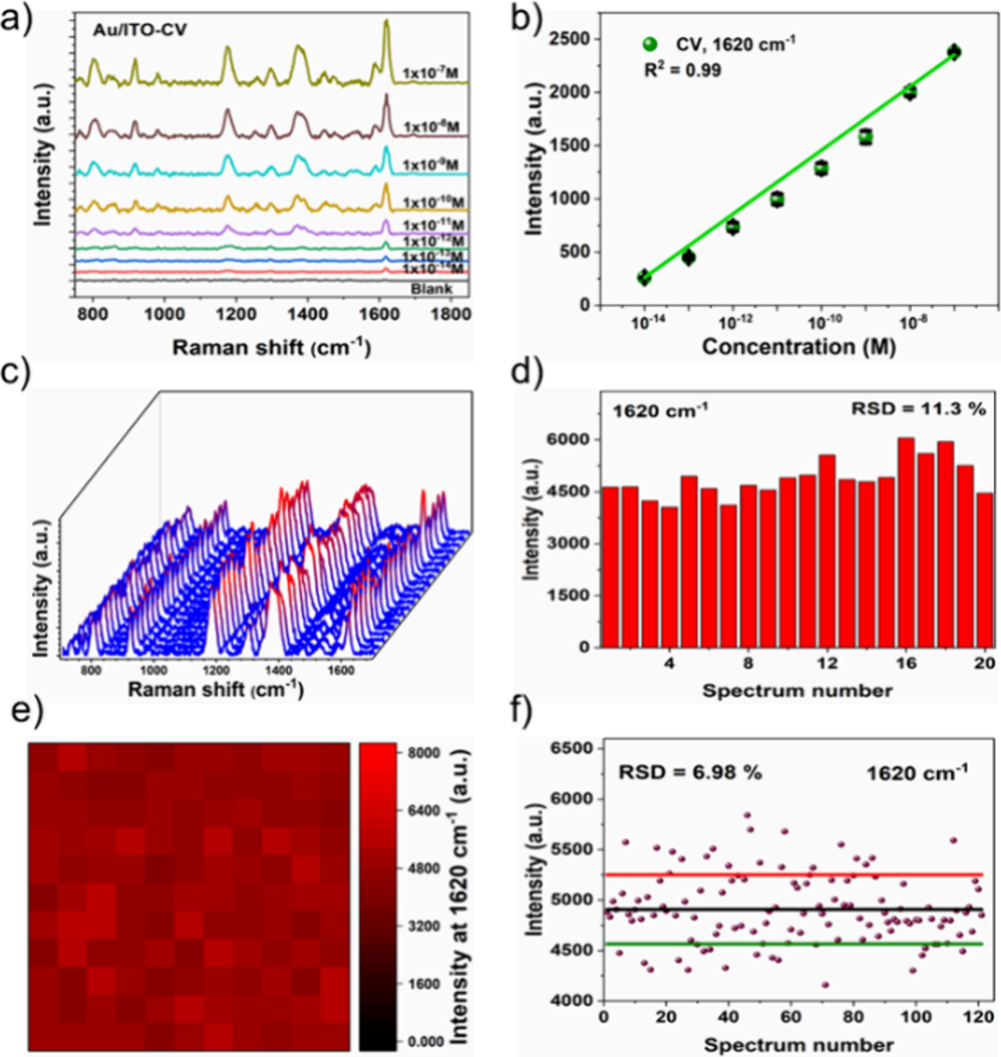
SERS performance of the Au/ITO substrates. (a, b) SERS spectra of CV with varied CV concentration and corresponding linear plot. (c) SERS spectra of CV (1 × 10^–6^ M) collected from 20 random spots on the porous Au/ITO substrates. (d) SERS intensity variation at the peak position of CV at 1620 cm^–1^ as a function of 20 different spots. (e) SERS mapping of 1620 cm^–1^ for CV (10^–6^ M) and (f) calculated SERS intensity changes at 1620 cm^–1^ from the SERS mapping.

To evaluate the SERS performance of the dendritic-shaped porous Au/ITO flexible substrate, CV (10^–6^ M) was used as a probe analyte molecule with a laser excitation wavelength of 633 nm. To evaluate the sensitivity of the Au/ITO substrate, different concentrations of CV solution ranging from 10^–6^ to 10^–14^ M were deposited onto the substrate and SERS spectra were recorded (Figure 2a). Figure 2a shows that the SERS spectra of CV display characteristic peaks at 806, 916, 1175, 1375, 1585, and 1620 cm^–1^, which correspond well with the CV molecules.^52^ Specifically, the peaks at 806 and 1172 cm^–1^ are attributed to the in-plane bending vibration of C–H, while the peaks at higher wavelengths, 1585 and 1620 cm^–1^, are ascribed to the stretching of ring C–C.^44,53^ Evidently, the Raman intensity exhibits a gradual decrease with the lowering CV concentration even at 10^–14^ M CV, and the characteristic peaks of CV were successfully detected, underlining the exceptional sensitivity of the flexible substrate. The limit of detection (LOD) is estimated according to the 3σ rule by using the following relation.^54^
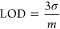
1where σ is the standard deviation of the SERS intensity of the blank measurements, while *m* represents the slope of plotted calibration curves. The calculated LOD of CV for the Au/ITO substrate is about 6.4 × 10^–15^ M, which is much greater compared with the many previously reported plasmonic SERS substrates. The derived calibration plot in Figure 2b displays a relationship between CV concentration vs Raman signal intensity at 1620 cm^–1^, following an almost a linear trend with slight deviations, which indicates that the substrate is capable of quantitative detection in complex real environments.

To verify the point-to-point reproducibility of the substrate, Raman spectra were acquired from 20 random spots within the same substrate (Figure 2c). As shown in Figure 2c,d, the SERS peak intensity of each spectrum at 1620 cm^–1^ of CV remains nearly identical, with a relative standard deviation (RSD) of about 11.3%, indicating excellent reproducibility of the porous Au/ITO flexible substrate. Notably, the calculated RSD values are lower than that of the SERS substrate based on colloidal monolayer Au NPs (RSD ≈ 15–20%).^55^ Moreover, as evidenced by the SERS mapping image, the color of the mapping at 1620 cm^–1^ of CV (10^–6^ M) is relatively uniform (Figure 2e). The relative peak intensity at 1620 cm^–1^ is almost similar to minimum deviation with the RSD of 6.98% recorded from a 2 × 2 μm^2^ area with a total of 121 spectra (Figure 2f), further demonstrating the excellent spectral uniformity of the as-fabricated dendritic Au/ITO substrate over a large area.

**Figure 3:**
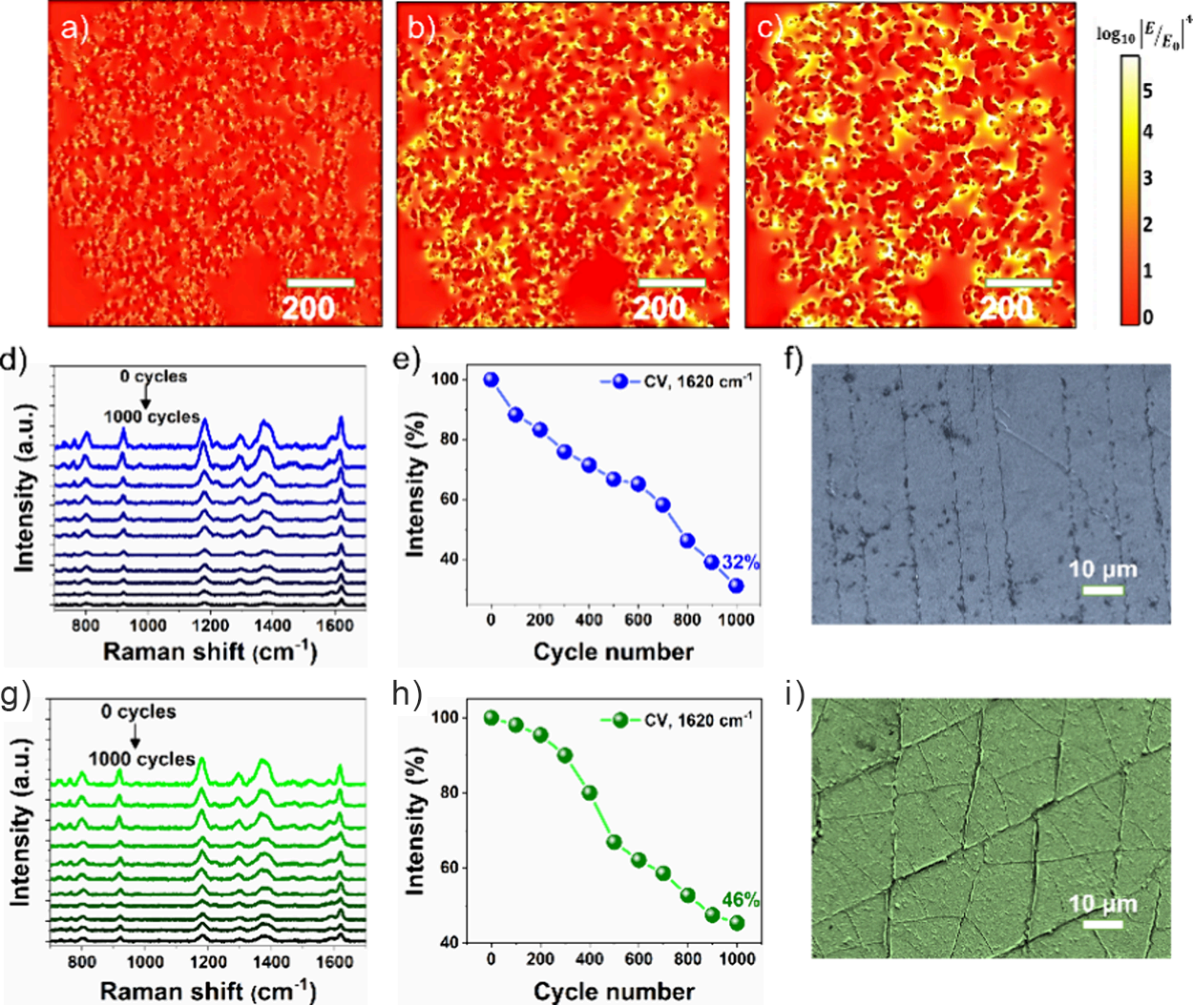
(a–c) FDTD simulation of the electromagnetic field distribution of dendritic Au films with three different laser excitation wavelengths of (a) 532, (b) 633, and (c) 785 nm. (d–i) Mechanical properties of the porous Au/ITO SERS substrate: (d, g) SERS spectra. (e, h) Corresponding variation of the SERS peak intensity of CV at 1620 cm^–1^ at different bending and twisting cycles. (f, i) Corresponding SEM images obtained after 1000 bending and twisting cycles.

To further investigate the density of “hotspots” in the dendritic Au/ITO flexible substrate for SERS enhancement, we carried out finite-difference time-domain (FDTD) simulations. Based on the SEM images in Figure 1a–c, the dendritic-like Au nanostructure with 3D morphology was chosen to estimate the electromagnetic (EM) field distributions. Figure 3a–c displays the estimated EM field distribution of the dendritic Au/ITO substrate under different laser excitation wavelengths of 532, 633, and 785 nm. Compared with the 532 nm excitation, the excitation wavelengths of 633 and 785 nm showed the strongest electromagnetic (EM) field distribution at the nanometric gaps between adjacent structures and the edges of the dendritic Au network, serving as SERS “hotspots”. The theoretical SERS enhancement factors (EFs) are estimated to be 3.6 × 10^3^, 4.8 × 10^5^, and 1.8 × 10^6^ for 532, 633, and 785 nm laser excitations, respectively. These results suggest that the excitation wavelengths of 633 and 785 nm showed high EM field distribution.

### Mechanical Properties of the 3D Au/ITO Flexible SERS Substrates

The mechanical robustness of the flexible substrates under different deformation conditions is a key parameter in their practical utilization. To determine the mechanical stability of the Au/ITO flexible substrate, the substrates were deposited with CV (10^–6^ M) and then subjected to mechanical stimuli such as bending with an angle of 75° and twisting to 135° for 1000 cycles and their SERS spectra were recorded (Figure 3d,g). As can be seen in Figure 3e,h, the SERS signal intensity of CV (10^–6^ M) at 1620 cm^–1^ on the Au/ITO flexible substrates was found to be reduced by about 32 and 46% after 1000 bending and twisting cycles, respectively, indicating appreciable mechanical stability. It is important to mention that the observed decrement in Raman signal intensities of CV after bending and twisting cycles could be attributed to the change in the self-assembly structure of dendritic Au networks over the ITO substrate, as few cracks can be observed in bending and twisting directions after 1000 cycles (Figure 3f,i). The AFM imaging results also further confirmed a similar observation that cracks formed in bending and twisting directions (Figure S5). Such formation of cracks can decrease the SERS hotspots, resulting in reduction of Raman signal intensities.

### SERS Detection of Nanoplastics Using a 3D Au/ITO Flexible Substrate

**Figure 4:**
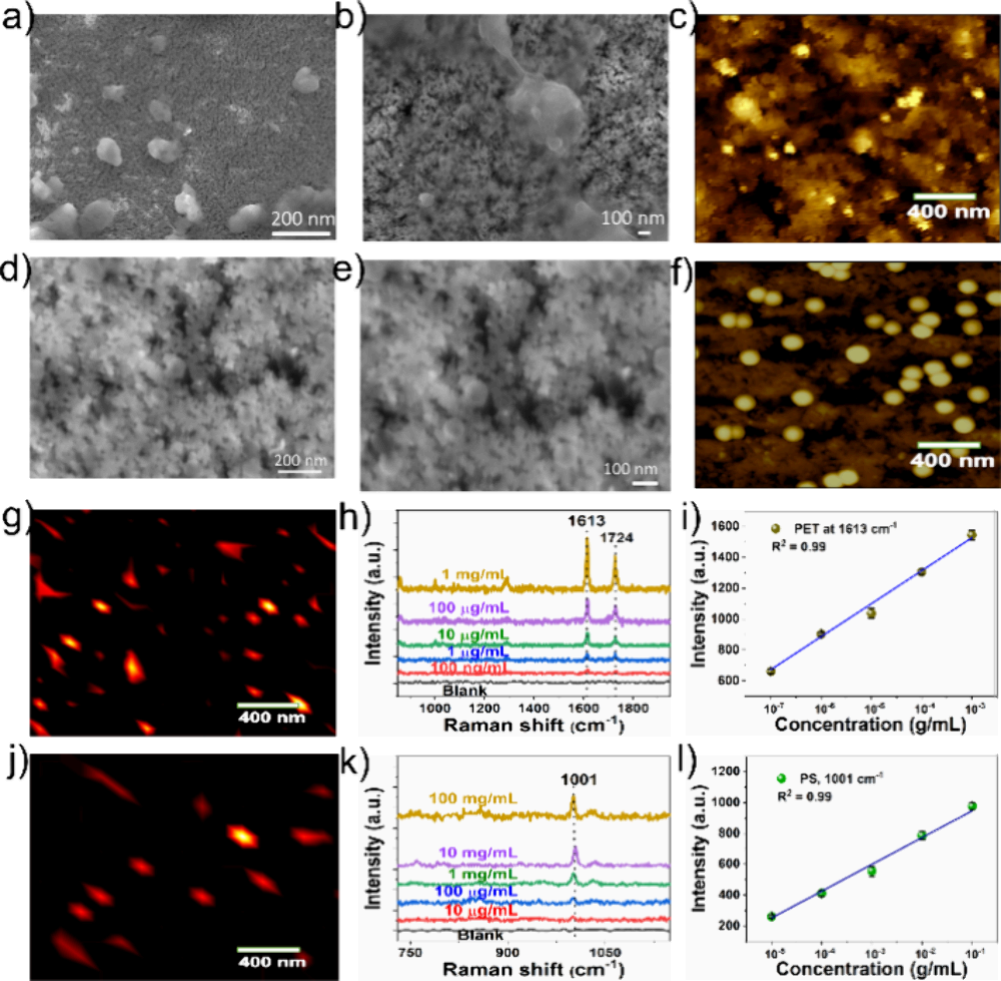
(a, b) SEM and (c) AFM topographic images of PET nanoplastics and (d, e) SEM and (f) AFM topographic images of PS nanoplastics over a flexible porous Au/ITO substrate. (g, j) Raman images of the PET and PS nanoplastics over the porous Au/ITO substrate. (h, k) SERS spectra of PET and PS nanoplastics with varied concentrations using the Au/ITO substrate and (i, l) corresponding linear calibration fit of the SERS peak intensity vs concentration of the PET and PS nanoplastics.

To test the applicability of the fabricated flexible Au/ITO substrates, we have selected detection of PET and PS nanoplastics, which are commonly present in environmental samples. Growing evidence indicates that micro- and nanoplastics are widely evidenced in drinking waters, milk products, food products, beverages, tea, etc.^56^ A majority of the studies indicated the presence of nanoplastics with a size range from 5 to ≥700 nm, where PET and PS nanoplastics were identified to be the most common.^57^ For PET nanoplastic detection, the PET nanospheres with a size of 200 nm were obtained from a drinking bottle by top-down fabrication,^50^ with the size and shape of given nanoparticles closer to what was expected in natural and complex environments. For comparison, the SERS detection of commercial PS nanoplastics (100 nm) was investigated. As evidenced from SEM and AFM topographic images (Figure 4a–f), the PET and PS spherical nanoplastic particles are uniformly dispersed and most of the particles are diffuse inside the pores in the porous Au/ITO flexible substrate. In addition, as noticed by the SERS mapping analysis (Figure 4g,j), the Raman intensity of PET and PS on porous Au/ITO is relatively uniform on the porous Au/ITO flexible substrate, further suggesting excellent SERS signal reproducibility.

To investigate the detection of nanoplastics and determine the LOD of the porous Au/ITO flexible substrate, SERS spectra were recorded with PET and PS nanoplastics with different concentrations (Figure 4h,k). As shown in Figure 4h, the SERS spectra of PET nanoplastics exhibited distinct peaks at 1288, 1613, and 1724 cm^–1^, corresponding to characteristic features of PET nanoplastics.^58^ Notably, the SERS signal was detected, even at a lower concentration of 100 ng/mL, indicating remarkable sensitivity. The LOD was estimated to be about 0.051 μg/mL, indicating ultralow LOD of the porous Au/ITO flexible substrate. Moreover, the linear behavior was observed for the concentration as a function of variation of the Raman peak intensity at 1613 cm^–1^ (Figure 4i), suggesting that the substrate is capable of trace detection of PET nanoplastics with excellent sensitivity. Moreover, the SERS spectra of PS nanoplastics (size of 100 nm) with varied concentrations (Figure 4k) showed a dominant peak at 1001 cm^–1^, which is associated with the C–C ring breathing vibration of PS nanospheres.^59^ The SERS spectra were clearly seen even at a concentration of 10 μg/mL, and the LOD was calculated to be about 8.2 μg/mL. Moreover, a linear trend was observed between PS concentrations as a function of the change in signal intensity of PS at 1001 cm^–1^ (Figure 4l). Notably, the signal intensity of PS nanoplastics is weaker, and a slightly low LOD value was observed compared with the PET nanoplastics, which could be due to the lower Raman scattering cross section of smaller PS nanoplastics, as observed in previous work.^51^ Importantly, the SERS detection performance of the porous Au/ITO flexible substrate is greater than those reported in the recent literature of plasmonic NP-based SERS substrates for nanoplastic detection (Table S1).

**Scheme 2:**
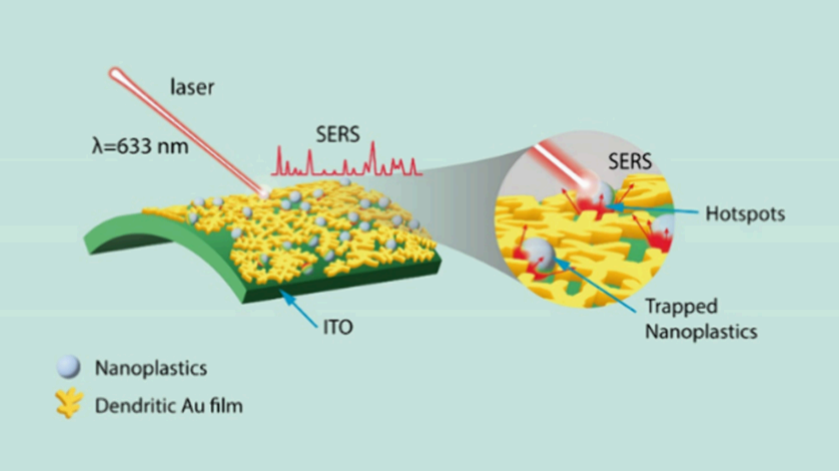
Schematic Illustration of the Proposed SERS Enhancement Mechanisms of the Dendritic Au/ITO Flexible Substrate for Nanoplastic Detection

Based on the above results, the SERS detection mechanism of the nanoplastics is schematically shown in Scheme 2. We hypothesized that the SERS enhancement process of a 3D dendritic Au/ITO substrate can be attributed to the following factors: (i) The 3D dendritic Au/ITO exhibits an LSPR peak at 618 nm that extends to near-infrared (NIR) regions (Figure S6), which can promote strong plasmonic coupling with the incident laser (λ = 633 nm), giving rise to intense electric distribution in the pore sites of the dendritic Au films as observed from FDTD simulation (Figure 3c). (ii) The interior pores within the 3D dendritic Au/ITO substrate allow effective diffusion and trapping of the smaller nanoplastic particles (<200 nm), thereby increasing the contact area between the nanoplastic particles and “hotspots”. Upon laser excitation, significant near-field SERS signal enhancement of the scattering cross section occurred due to intense electric field distribution in the “hotspots” of the dendritic Au/ITO substrate (Scheme 2).^26,60,61^ In contrast, PET nanoplastics coated onto a flat Au film and Au NP-deposited ITO substrates produced lower SERS signal intensities (Figure S7), indicating the presence of poor “hotspots”. Moreover, nanoplastics supported over a bare ITO substrate exhibited negligible SERS signals under the same conditions (Figure S8). (iii) The larger nanoplastics (>200 nm) that are placed within a distance of <10 nm proximity over the surface of the dendritic Au/ITO substrate can also enhance the far-field intensities due to their interaction with the Au surface and nearby “hotspots”.^27^ Therefore, the enhancement effect of nanoplastics in the 3D Au/ITO substrate depends on the position and size, as proposed by several previous studies.^26,27,46^

### Detection of PET Nanoplastics in Real Environmental Samples

**Figure 5:**
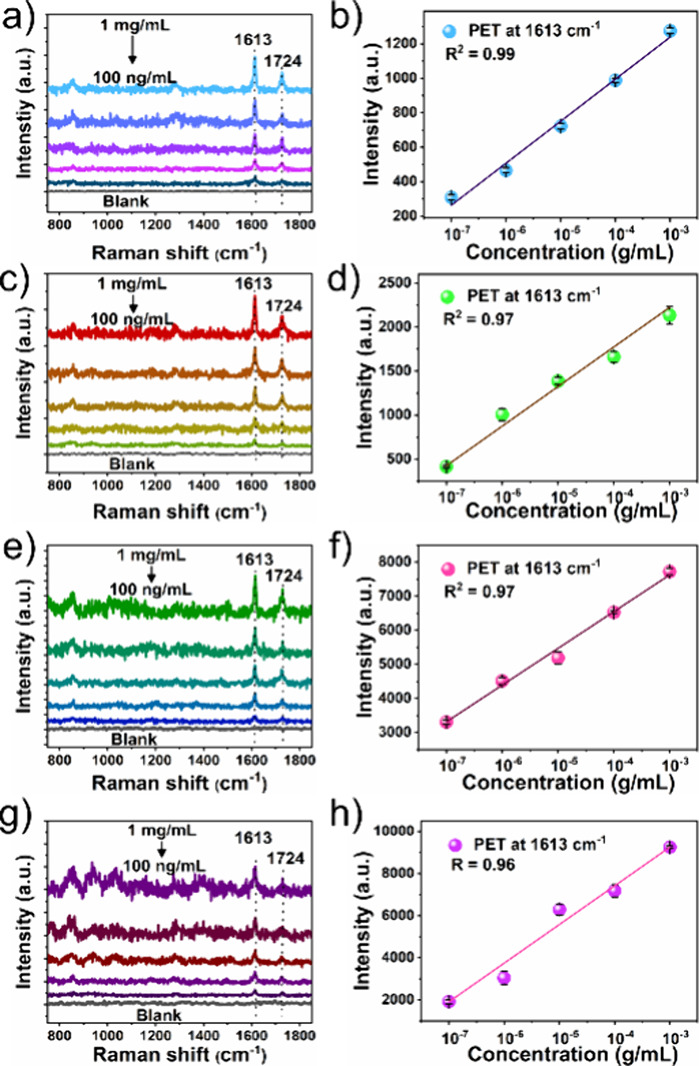
SERS spectra of the PET nanoplastic particles with varied concentrations and linear calibration plots of the self-assembled porous Au NP film (Au-P4) substrate in water and milk environments. (a, b) Tap water, (c, d) lake water, (e, f) diluted milk, and (g, h) diluted wine.

The precise detection of nanoplastics in a real environmental medium remains an imposing challenge due to the difficulties and interference of environmental contaminations. To demonstrate the applicability of the flexible Au/ITO substrate in SERS detection of PET nanoplastics in complex environmental water samples and beverages, we explored four types of samples (tap water, lake water, diluted milk, and wine). Figure 5a–h presents the SERS spectra of the control sample and PET nanoplastics with various concentrations spiked in tap water, lake water, diluted milk, and wine onto the porous Au/ITO substrate and corresponding linear calibration plots. The results demonstrated that the SERS substrate effectively detects the PET NPs in tap water, lake water, diluted milk, and wine, and the SERS signal was detected even at lower concentrations of 1, 100, 100, and 10 ng/mL. The LODs were estimated to be about 0.066, 0.08, 0.084, and 0.96 μg/mL for tap water, lake water, diluted milk, and wine sample, respectively. For comparison, four real samples (without spiking of PET nanoplastics) were deposited over the Au/ITO substrate and SERS spectra were recorded. The results showed that no peaks were detected in four real samples (Figure S9), indicating that there are no detectable concentrations of nanoplastics tested in real samples. These findings indicate that the porous Au/ITO substrates exhibit remarkably high sensitivity in detecting PET NPs in real environmental samples. In addition, a flexible Au/ITO substrate also demonstrated high sensitivity in detecting PS nanoplastics in real environmental samples such as tap water and lake water (Figure S10). Thus, the as-fabricated porous Au/ITO SERS substrate showcases exceptional sensitivity for quantitative SERS detection of various nanoplastics in complex environmental samples.

## Conclusions

In summary, we have demonstrated a facile, low-cost strategy for the fabrication of large-area, highly flexible, and efficient 3D SERS substrates for sensitive detection of nanoplastics. The porous Au NP films with uniform porosity and high density of “hotspots” onto a flexible ITO substrate were achieved through interfacial self-assembly of thermally evaporated Au onto the DES surface and subsequent transfer process over the ITO surface. The as-prepared dendritic Au/ITO substrate exhibits high sensitivity for detecting the analyte molecule (CV) with a LOD as low as 6.4 × 10^–15^ M and exceptional signal reproducibility with a lower RSD value of 11.3%. In addition, the SERS substrates showed exceptional mechanical flexibility by retaining about 32 and 46% of their SERS activity even after 1000 bending and twisting cycles, respectively. Importantly, the prepared flexible substrate can be used for SERS detection of PET and PS nanoplastics with LODs of 0.051 and 8.2 μg/mL, respectively. Thus, our simple and low-cost approach opens up new avenues for the large-area fabrication of highly flexible plasmonic substrates for SERS detection, including monitoring of pesticides, virus contamination, a variety of environmental pollutants, and drugs.

## Supporting Information Available

Photographic images of Au NP deposition over the DES-coated glass surface, SEM images of the Au film, SEM images of pore size distribution of dendritic Au and Au NP films, AFM topographic images after 1000 bending and twisting cycles, UV–vis absorption spectra, comparison of SERS spectra of PET nanoplastics, SERS mapping images, and SERS spectra of nanoplastics in real samples (PDF)
