# Supplementary material for: Mechanically Flexible, Large-Area Fabrication of Three-Dimensional Dendritic Au Films for Reproducible Surface-Enhanced Raman Scattering Detection of Nanoplastics
Source: ACS Sens. 2024 Oct 31;10(3):1747–55. doi: 10.1021/acssensors.4c02081 (PMC11959597; doi:10.1021/acssensors.4c02081)
Supplement: Supplementary file 1 — se4c02081_si_001.pdf [file se4c02081_si_001.pdf]

<u>**Supporting information for:**</u>

Mechanically Flexible, Large-Area Fabrication of 3D dendritic Au Films for Reproducible SERS-Detection of Nanoplastics

Rafael Villamil Carreón, [†] Ana G. Rodríguez-Hernández,[ǁ] Laura Elvira Serrano de la Rosa, [$] José Juan Gervacio-Arciniega[§,] Siva Kumar Krishnan [#,] *

[†]Facultad de Ciencias Físico Matemáticas, Benemérita Universidad Autónoma de Puebla, Av. San Claudio y Av. 18 sur., Puebla, Pue., C. P. 72570, México.

[ǁ]CONAHCyT-Centro de Nanociencias and Nanotecnología, Universidad Nacional Autónoma de México, Km 107 Carretera Tijuana-Ensenada. Ensenada, C.P.22800 Baja California, México.

[$]Instituto de Física, Benemérita Universidad Autónoma de Puebla, Apdo. Postal J-48, Puebla, Pue. 72570, México.

[§]CONAHCyT- Facultad de Ciencias Físico Matemáticas, Benemérita Universidad Autónoma de Puebla, Apdo. Postal J-48, Puebla 72570, México.

[#]CONAHCyT-Instituto de Física, Benemérita Universidad Autónoma de Puebla, Apdo. Postal J-48, Puebla, Pue. 72570, México.

Corresponding author:

SKK: sivakumar@ifuap.buap.mx

Table of contents:

Figure S1. Photographic image of (a) DES (ChCl: urea, molar ratio of 1:2) coated glass substrate (b) Au-NPs deposited onto the surface of DES during thermal evaporation process……………………………………………………………………………………..**S3**

Figure S2. SEM images of Au films obtained without utilizing DES onto growth substrate (Glass)……………………………………………………………………………......**S4**

Figure S3. SEM image, histogram of particles size distribution and pore-size of dendritic Au NPs films………………………………………………………………………………**S5**

Figure S4. Low and high magnification FE-SEM images of self-assembled Au NPs obtained at higher deposition pressure ($1x10^{-2}$ mbar)……………………………………………….**S6**

Figure S5: AFM topographical image of Au/ITO flexible substrate after bending, twisting over 1000-cycles, respectively……………………………………………………………..**S7**

Figure S6: UV-vis spectra of Au NPs film (thermally deposited at $1x10^{-2}$ mbar), and 3D dendritic Au film (deposited at $2x10^{-4}$ mbar), respectively………………………………..**S8**

Figure S7. Comparison of the SERS spectra of PET nanoplastics particles over 3D dendritic Au/ITO substrate with the flat Au film, and Au NPs films, respectively…………………**S8**

Figure S8. SERS spectra and SERS-mapping images of PET and PS nanoplastics onto the bare ITO flexible substrate (without dendritic Au NPs films on it)……………………….**S9**

Figure S9: SERS spectra of four different real-samples onto the Au/ITO flexible substrate (without spiking of PET nanoplastics) …………………………………………………**S10**

Figure S10. SERS spectra of PS nanoplastics (100 nm) dispersed in a) tap water, b) lake water onto the Au/ITO flexible substrate…**………………………………………………S11**

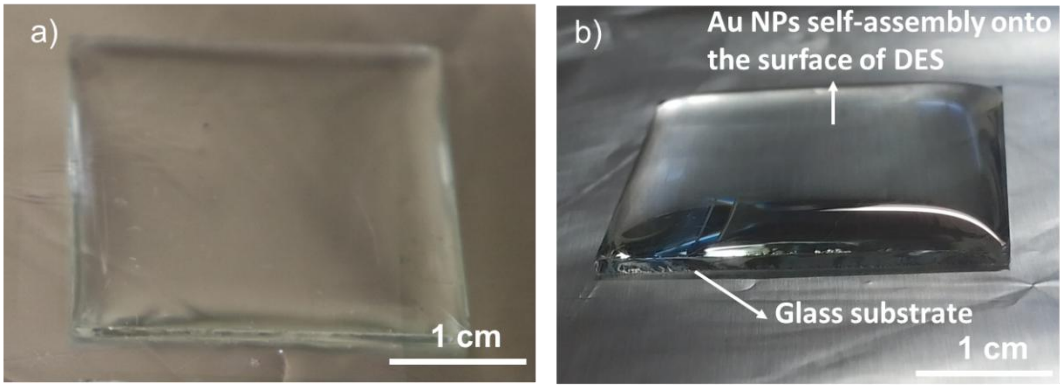

Figure S1. Photographic image (Top view) of a) DES (ChCl: urea, molar ratio of 1:2) coated glass substrate b) Au-NPs films deposited onto the surface of DES using vacuum thermal evaporation process.

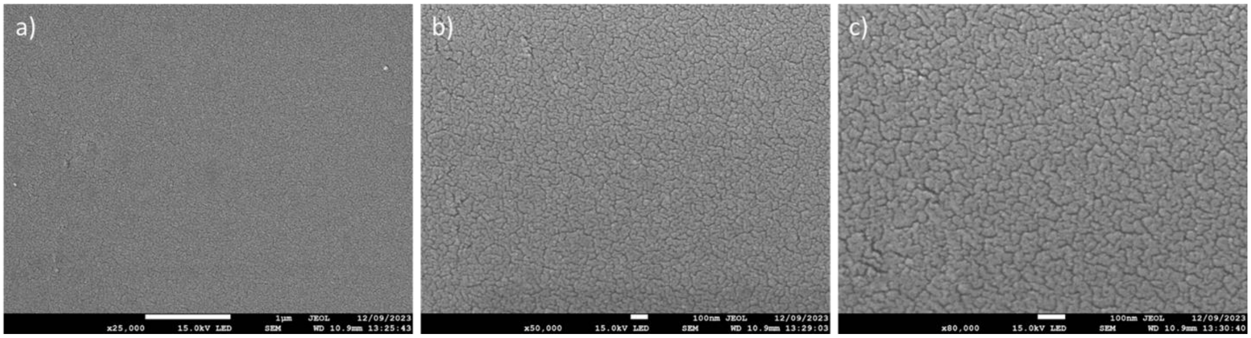

Figure S2. a-c) Typical SEM images of Au films obtained without utilizing DES onto growth substrate (glass). Deposition pressure of $2x10^{-4}$ mbar, applied current of 4 amperes.

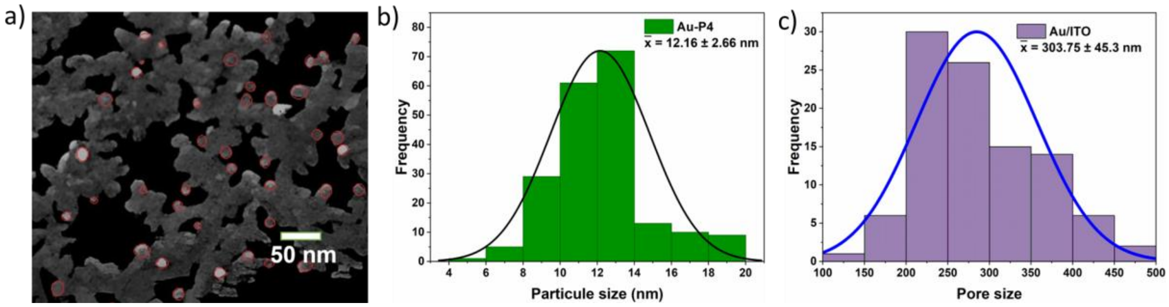

Figure S3. a) Magnified SEM image of dendritic Au NPs films, b) histogram of particles size distribution, c) pore-size distribution histogram, respectively.

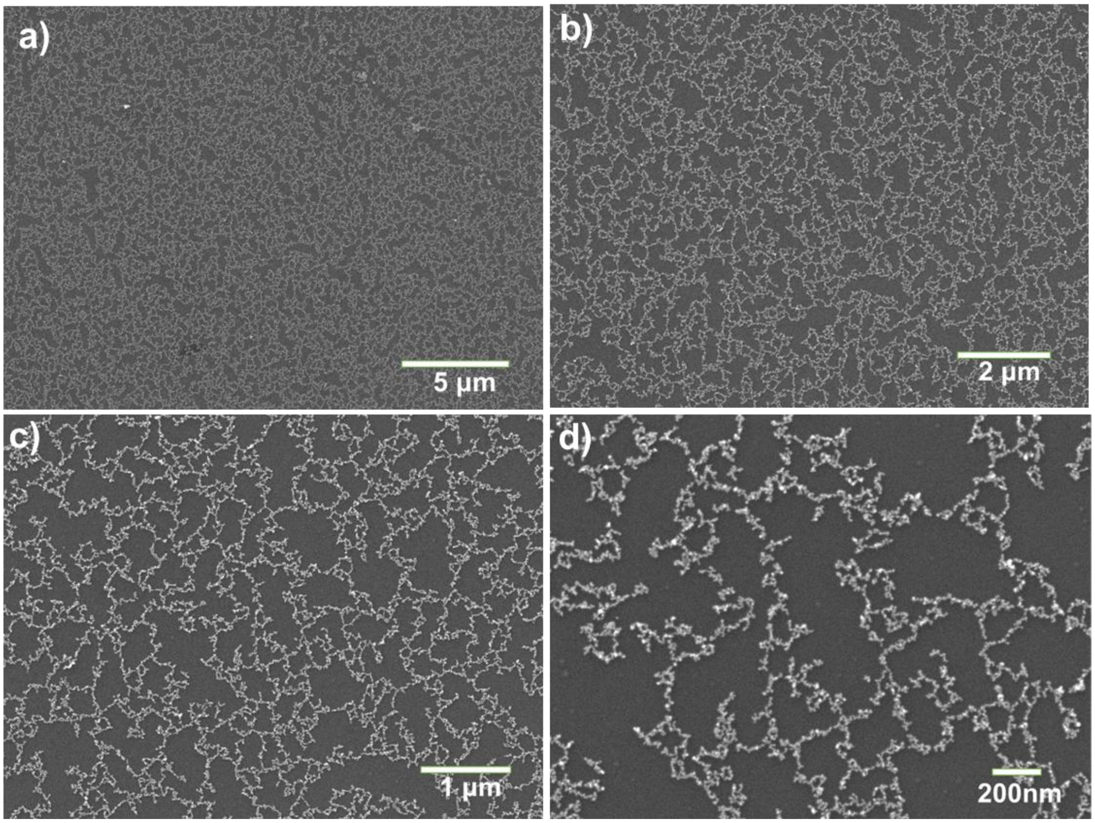

Figure S4. a, b) low and c, d) high magnification FE-SEM images of self-assembled Au NPs obtained at higher deposition pressure ($1x10^{-2}$ mbar).

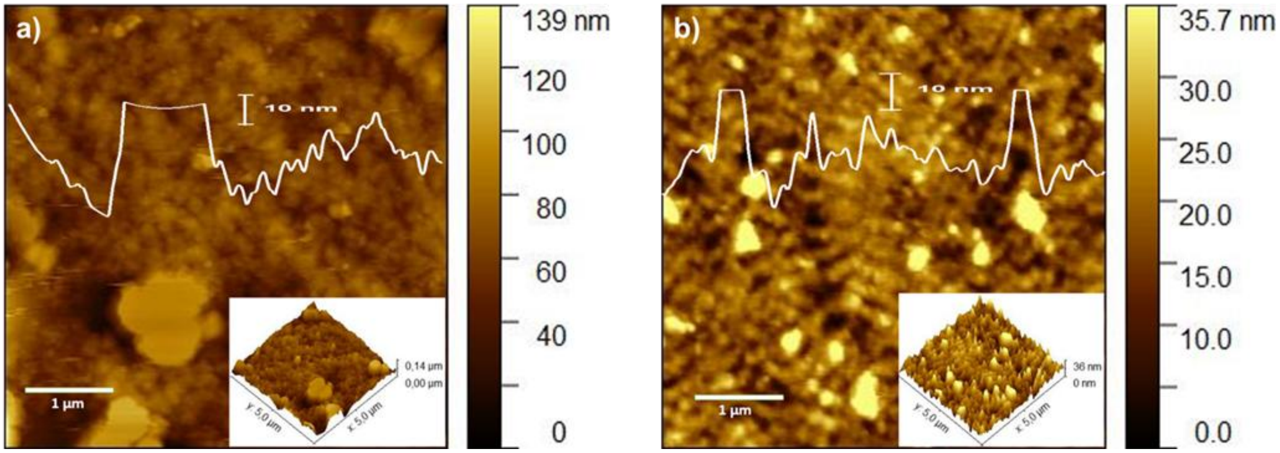

Figure S5: AFM topographical image of Au/ITO flexible substrate after a) bending, b) twisting over 1000-cycles, respectively.

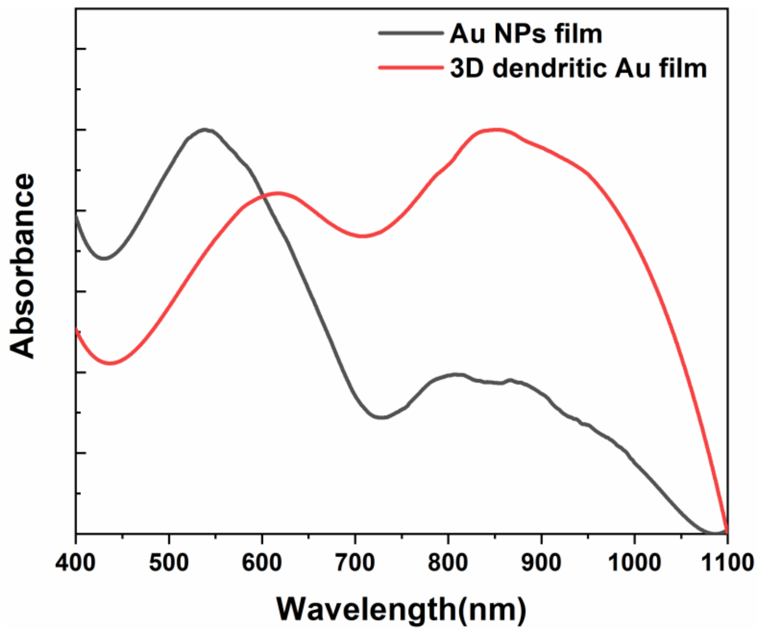

Figure S6. UV-vis spectra of Au NPs film (thermally deposited at 1x10^{-2} mbar), and 3D dendritic Au film (deposited at 2x10^{-4} mbar), respectively.

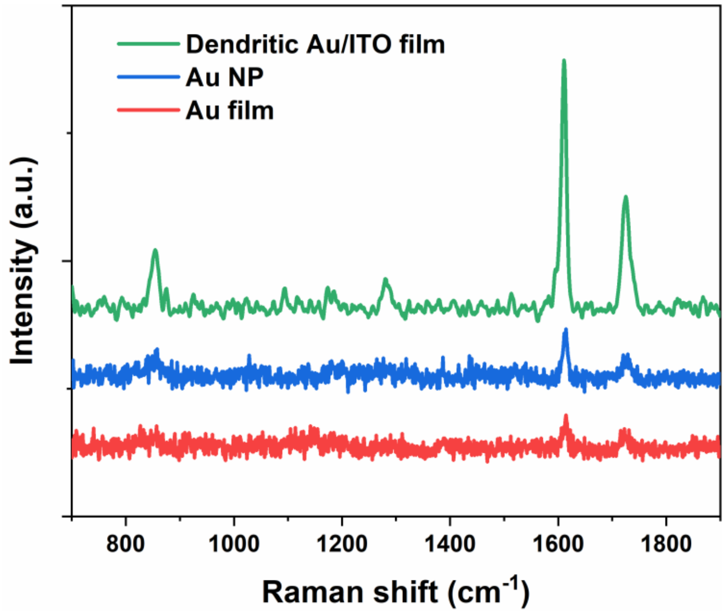

Figure S7. Comparison of the SERS spectra of PET nanoplastics particles over 3D dendritic Au/ITO substrate with the flat Au film, and Au NPs films, respectively.

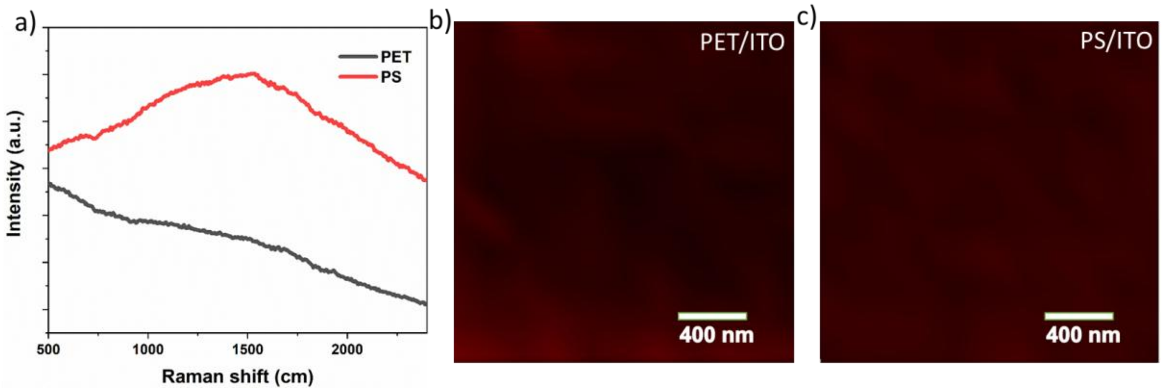

Figure S8: a) SERS spectra, b, c) SERS-mapping images of PET and PS nanoplastics onto the bare ITO flexible substrate (without dendritic Au NPs films on it).

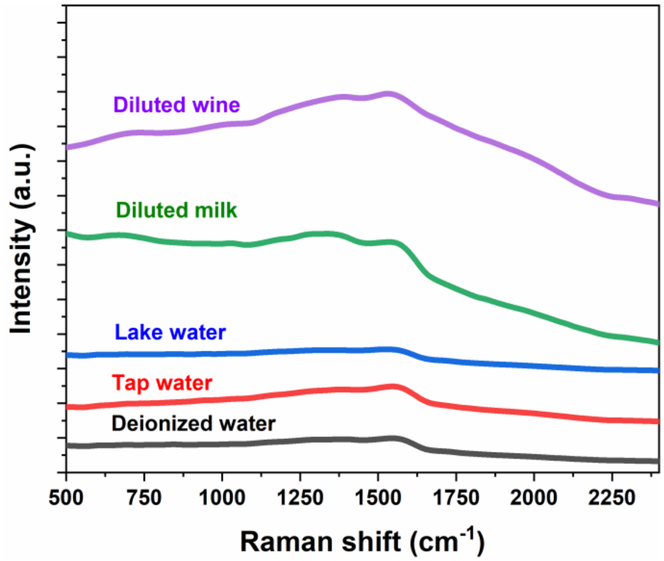

Figure S9: SERS spectra of four different real-samples onto the Au/ITO flexible substrate (without spiking of PET nanoplastics).

Table S1. Comparison performance of the SERS-detection parameters for dendritic Au/ITO flexible substrate for nanoplastics detection.

SERS substrate	Type of nanoplastics	Sensitivity	nanoplastic Size	References
Ag/ZnO@PDMS	Polystyrene	25 µg/mL	800 nm	Zhau et al.,[1]
Au NPs	Polystyrene	6.5 µg/mL	1-4 µm	Mikac et al.,[2]
Au pyramidal cavities	Polystyrene<br>PMMA	26.3 µg/mL<br>26.3 µg/mL	360 nm<br>500 nm	Xu et al.,[3]
Au NPs decorated sponge	4-mercaptopyridine	0.05 mg/L	39-155 µm	Yin et al.[4]
AgNPs–$MgSO_4$	Polystyrene	100, 100 µg/mL	50 nm, 1µm	Zhou et al.,[5]
AuNSs@Ag@AAO	Polystyrene	50 µg/mL	400 nm	Le tal.,[6]
Porous Au NPs film	polyethylene terephthalate	1 µg/mL	50-300 nm	this work

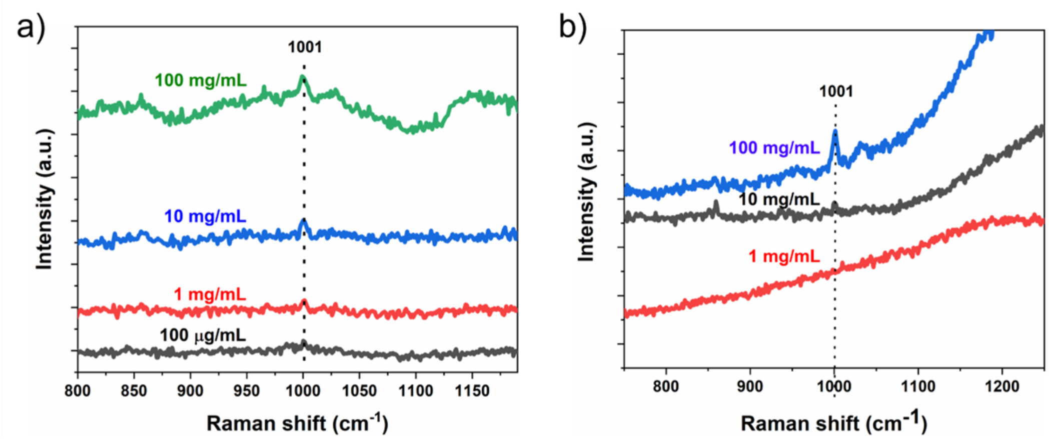

Figure S10. SERS spectra of PS nanoplastics (100 nm) dispersed in a) tap water, b) lake water onto the Au/ITO flexible substrate.

References:

(1) Zhu, Z.; Han, K.; Feng, Y.; Li, Z.; Zhang, A.; Wang, T.; Zhang, M.; Zhang, W. Biomimetic Ag/ZnO@PDMS Hybrid Nanorod Array-Mediated Photo-Induced Enhanced Raman Spectroscopy Sensor for Quantitative and Visualized Analysis of Microplastics. *ACS Appl Mater Interfaces* **2023**, *15*, 36988–36998.

(2) Mikac, L.; Rigó, I.; Himics, L.; Tolić, A.; Ivanda, M.; Veres, M. Surface-Enhanced Raman Spectroscopy for the Detection of Microplastics. *Appl Surf Sci* **2023**, *608*.

(3) Xu, G.; Cheng, H.; Jones, R.; Feng, Y.; Gong, K.; Li, K.; Fang, X.; Tahir, M. A.; Valev, V. K.; Zhang, L. Surface-Enhanced Raman Spectroscopy Facilitates the Detection of Microplastics <1 Mm in the Environment. *Environ Sci Technol* **2020**, *54* (24), 15594–15603.

(4) Yin, R.; Ge, H.; Chen, H.; Du, J.; Sun, Z.; Tan, H.; Wang, S. Sensitive and Rapid Detection of Trace Microplastics Concentrated through Au-Nanoparticle-Decorated Sponge on the Basis of Surface-Enhanced Raman Spectroscopy. *Environmental Advances* **2021**, *5*, 100096.

(5) Zhou, X. X.; Liu, R.; Hao, L. T.; Liu, J. F. Identification of Polystyrene Nanoplastics Using Surface Enhanced Raman Spectroscopy. *Talanta* **2021**, *221*, 121552.

(6) Lê, Q. T.; Ly, N. H.; Kim, M. K.; Lim, S. H.; Son, S. J.; Zoh, K. D.; Joo, S. W. Nanostructured Raman Substrates for the Sensitive Detection of Submicrometer-Sized Plastic Pollutants in Water. *J Hazard Mater* **2021**, *402*,123499.
